# Blood Gases, Biochemistry, and Hematology of Galapagos Green Turtles (*Chelonia Mydas*)

**DOI:** 10.1371/journal.pone.0096487

**Published:** 2014-05-13

**Authors:** Gregory A. Lewbart, Maximilian Hirschfeld, Judith Denkinger, Karla Vasco, Nataly Guevara, Juan García, Juanpablo Muñoz, Kenneth J. Lohmann

**Affiliations:** 1 North Carolina State University, College of Veterinary Medicine, Raleigh, North Carolina, United States of America; 2 University San Francisco de Quito, Galapagos Science Center, Puerto Baquerizo Moreno, Galapagos, Ecuador; 3 Galapagos National Park Service, Puerto Ayora, Galapagos, Ecuador; 4 Department of Biology, University of North Carolina, Chapel Hill, North Carolina, United States of America; Ecole Normale Supérieure de Lyon, France

## Abstract

The green turtle, *Chelonia mydas,* is an endangered marine chelonian with a circum-global distribution. Reference blood parameter intervals have been published for some chelonian species, but baseline hematology, biochemical, and blood gas values are lacking from the Galapagos sea turtles. Analyses were done on blood samples drawn from 28 green turtles captured in two foraging locations on San Cristóbal Island (14 from each site). Of these turtles, 20 were immature and of unknown sex; the other eight were males (five mature, three immature). A portable blood analyzer (iSTAT) was used to obtain near immediate field results for pH, lactate, pO_2_, pCO_2_, HCO_3_^−^, Hct, Hb, Na, K, iCa, and Glu. Parameter values affected by temperature were corrected in two ways: (1) with standard formulas; and (2) with auto-corrections made by the iSTAT. The two methods yielded clinically equivalent results. Standard laboratory hematology techniques were employed for the red and white blood cell counts and the hematocrit determination, which was also compared to the hematocrit values generated by the iSTAT. Of all blood analytes, only lactate concentrations were positively correlated with body size. All other values showed no significant difference between the two sample locations nor were they correlated with body size or internal temperature. For hematocrit count, the iSTAT blood analyzer yielded results indistinguishable from those obtained with high-speed centrifugation. The values reported in this study provide baseline data that may be useful in comparisons among populations and in detecting changes in health status among Galapagos sea turtles. The findings might also be helpful in future efforts to demonstrate associations between specific biochemical parameters and disease.

## Introduction

The green turtle (*Chelonia mydas*), also known as the black turtle in the Pacific Ocean, is a marine chelonian inhabiting oceans throughout the world [57]. The green turtle is currently listed as Endangered on the IUCN Red List, and no commercial use is permitted under CITES Appendix I. Major threats to green turtle populations include habitat destruction, pollution, disease, consumption of meat and eggs by local populations, fishing gear entanglement, and consumption of plastics and other anthropogenic materials [16], [37], [42], [43], [59]. Health assessments of green turtles may therefore have implications for wildlife biology and species conservation. Considerable research on natural history has been performed in this species and studies on the health parameters of green turtles, while still relatively limited, have increased dramatically in the last 5 years [1], [4], [5], [8], [17], [18], [22], [27], [30], [31], [39], [40], [45], [50]. A recent review summarizes the health of wild sea turtles and methods of assessment, including blood parameters [20].

Biochemical and hematology parameters, as measured in peripheral blood, are a useful diagnostic tool in animal health management [2], [3]. To determine the significance of alterations in biochemical and hematological values, it is essential to establish species-specific (or at least taxon-specific) normal values for parameters of interest. Reference intervals for certain chelonian species, including sea turtles [2], [5], [6], [8]–[10], [12], [15], [18], [19], [24], [33], [34], [39], [46], [60], have been widely investigated. Additionally, wild sea turtle blood gas values have been reported and analyzed [28], [55]. However, few studies have combined blood gas, biochemistry, and hematology parameters from the same geographic subpopulation of sea turtles. The present study evaluates selected blood gas, blood biochemical, and hematology parameters from 28 wild-caught green turtles (*Chelonia mydas*) in two coastal foraging areas adjacent to San Cristóbal Island, Galapagos, Ecuador. The current study appears to be the first health assessment study on Galapagos sea turtles and on Western Hemisphere green turtles south of the Equator.

## Materials and Methods

### Ethics Statement

This study was performed as part of a population health assessment approved and supported by the Galapagos National Park Service (Permit # PC-35-12 to J. Denkinger) and approved by the Universidad San Francisco de Quito ethics and animal handling protocol. All handling and sampling procedures were consistent with standard vertebrate protocols and veterinary practices.

### Turtle Capture and Sampling

Turtles were captured in shallow water within 100 m from the shore by swimmers using standard snorkel, mask, and fins. The animals were carried to shore and placed on the beach. Turtles were aligned with their heads facing the water so that the slight incline of the beach assisted efforts to obtain blood samples from the dorsal jugular vein. A few turtles were sampled while still partially in water when a receding tide exposed rocks, which prevented turtles from being carried safely onto the sandy beach.

Blood samples were obtained within approximately 5 minutes of capture. Fourteen turtles were captured, examined, and sampled from La Loberia Beach (0° 55′ 40″ S, 89° 36′ 43″ W) on 30 June, 2013 and 14 turtles were similarly captured and sampled on 1 July, 2013 at a second site at Punta Carola (0° 53′ 26″ S, 89° 34′ 46″ W). Both study sites are shallow bays of similar size, sheltered from wave exposure, and were identified as important foraging and resting areas where individual turtles show very high site fidelity [16]. To avoid capturing the same individual more than once, a line of white zinc oxide ointment was applied to each turtle’s carapace after a blood sample had been obtained and before the animal was released. The line was clearly visible underwater and remained on for at least a day, but disappeared soon after (Hirschfeld, personal observation).

In addition to obtaining a blood sample from each turtle, photographs were taken of each side of the head, as well as the carapace. These images can be used to identify individuals in the future [35], [44], [47].

### Blood Sample Collection and Handling

All turtles were manually restrained and blood samples of approximately 2.5 mLs were obtained from either the left or right dorsal jugular sinus using a heparinized 22 gauge needle attached to a 3.0 mL syringe. The blood was then immediately divided into sub-samples. Some subsamples were used for making blood films on clean glass microscope slides, some were stored on ice in sterile plastic vials for future analyses, and others were loaded into the CG-8+ and CG-4+ iSTAT cartridges within 10 minutes of sample collection.

### Blood Gas and Biochemistry Parameters

**Figure 1:**
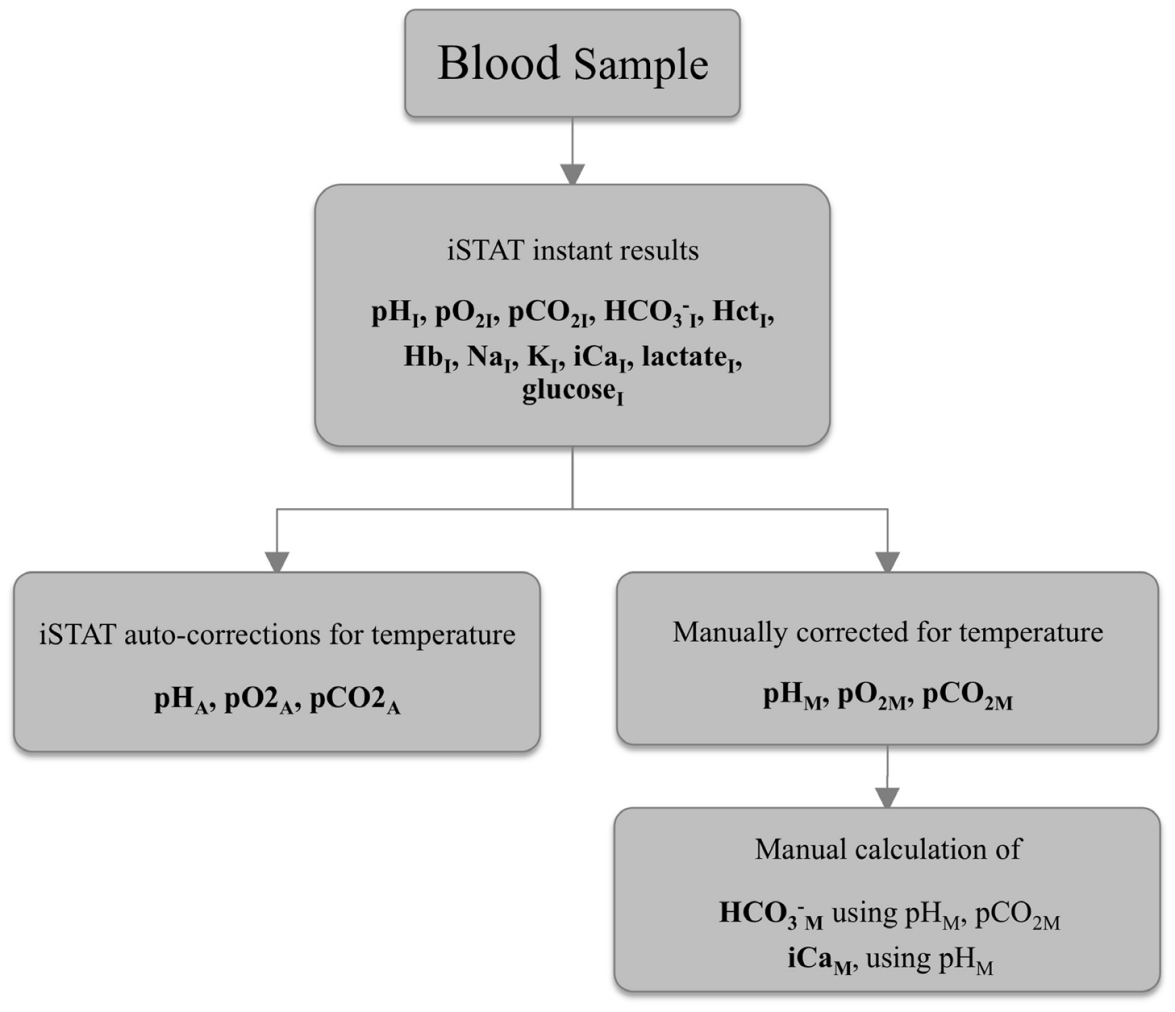
Flow chart of the iSTAT sample handling and calculations.

The blood gas, electrolyte, and biochemistry results were obtained using an iSTAT Portable Clinical Analyzer (Heska Corporation, Fort Collins, Colorado, USA) with CG8+ and CG4+ cartridges. The iSTAT is a portable, handheld, battery-operated electronic device with the ability to measure a wide variety of blood gas, chemistry, and basic hematology parameters with only a few drops (0.095 mL) of whole, non-coagulated blood. The following parameters were measured and recorded: pH, lactate, pO_2,_ pCO_2_, HCO_3_^−^, Hct, Hb, Na, K, iCa, and glucose. The iSTAT device analyzed the blood at 37°C then corrected pH, pO_2_, and pCO_2_ for body temperature once this information was entered. Because the validity of the iSTAT temperature corrections has been questioned by some authors [13], [28], we also manually calculated an independent set of corrections for pH, pO_2_, pCO_2_, iCa, and HCO_3_^−^ based on the turtle cloacal temperature (Ti) at the time of sampling [5], [38], using the equations listed below. Both sets of values (i.e., those derived from auto-corrections from the iSTAT and those derived from independent calculations) are reported in Table 1. The methodology for obtaining the various measured and calculated values is summarized in Figure 1. For clarity, instant values obtained from the iSTAT are denoted by an ‘I’ subscript. Those that were auto-corrected for temperature by entering the turtle’s body temperature into the iSTAT are denoted by ‘A’ subscript. Values that were manually corrected for temperature using the equations shown below are denoted by ‘M’ subscript.

**Table 1:** Mean, standard deviation, and range for blood gas and blood biochemical values for wild Galapagos green turtles.

Analyte	n	Mean	SD	Min	Max
HCO_3_^−^_I_ (mmol/L)	24	43.8	5.8	35.6	58.2
HCO_3_^−^_M_ (mmol/L)	28	41.1	5.6	33.0	54.4
pH_A_	28	7.477	0.085	7.273	7.626
pH_M_	28	7.441	0.075	7.256	7.568
pCO_2A_ (mmHg)	24	46.5	7.1	32.4	65.4
pCO_2M_ (mmHg)	28	49.0	9.2	32.4	68.3
pO_2A_ (mmHg)	28	22	5	14	32
pO_2M_ (mmHg)	28	53	10	36	72
Na_I_ (mmol/L)	28	148	3	143	153
K_I_ (mmol/L)	28	3.4	0.5	2.7	4.3
iCa_I_ (mmol/L)	27	0.87	0.14	0.64	1.18
iCa_M_ (mmol/L)	27	0.79	0.12	0.57	1.06
Glu_I_(Umg/dl)	28	60	9	46	82
Hct_I_(L/L)	28	0.24	0.05	0.17	0.38
Hb_I_ (g/L)	28	80	16	58	129
Lac_I_ (mmol/L)	28	3.73	2.44	0.8	8.73

‘I’ subscript denotes values obtained through the instant iSTAT analysis, ‘M’ subscript indicates values manually corrected for temperature using standard equations (see Results and Figure 1), and ‘A’ subscript indicates values that were auto-corrected for temperature by the iSTAT after a turtle’s cloacal temperature was entered into the iSTAT.



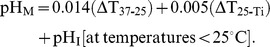



Anderson et al. (2011) [5] simplifies the above for Ti values below 25°C:


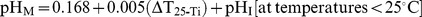
. (We note that a typographical error in [5] shows 0.0168 instead of 0.168.).

Because pH has an effect on iCa calculation, the formula below was used in the current study [5], [21], [32]:
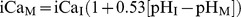



The following formulas [5] were used to correct pCO_2_ and pO_2_ for temperature:
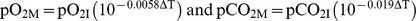



For the calculation of bicarbonate the following adaptation of the Henderson-Hasselbach Equation was used [36], [51]:
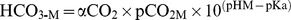



The corresponding αCO_2_ and pKa values were calculated using the formulas published by Stabenau and Heming (1993) [51].

### Hematology

Heparinized whole blood was stored on ice immediately after collection and refrigerated overnight. Total erythrocyte and leukocyte counts (Table 2) were obtained within 24 hours using Natt Herick’s stain and a Neubaeuer hemocytometer [11]. Hematocrit was determined using high-speed centrifugation of blood-filled microhematocrit tubes. Differential white blood cell counts were conducted by examining 100 white blood cells on a peripheral smear stained with Wright-Giemsa stain.

**Table 2:** Mean, standard deviation, and range for manually analyzed hematology values of wild Galapagos green turtles.

Analyte	n	Mean	SD	Min	Max
Hct (L/L)	28	0.236	0.048	0.17	0.38
WBC (x10^9^/L)	28	6.58	4.02	1.76	22.4
Heterophils (%)	28	16.4	6.6	8.0	35.0
Lymphocytes (%)	28	50.5	7.72	33.0	67.0
Monocytes (%)	28	12.0	5.73	6.0	32.0
Azurophils (%)	28	0.04	0.19	0	1.0
Eosinophils (%)	28	20.8	5.99	5.0	31.0
Basophils (%)	28	0.36	0.56	0	2.0
Heterophils (x10^9^/L)	28	1.06	0.66	0.23	2.59
Lymphocytes (x10^9^/L)	28	3.42	2.24	0.97	119
Monocytes (x10^9^/L)	28	0.79	0.60	0.18	2.92
Azurophils (x10^9^/L)	28	0.001	0.01	0	0.03
Eosinophils (x10^9^/L)	28	1.41	1.07	0.37	5.16
Basophils (x10^9^/L)	28	0.02	0.04	0.00	0.13

### Turtle Measurements and Body Temperature

A flexible measuring tape was used to determine curved carapace lengths (CCL). The sex of an immature sea turtle cannot be determined with confidence on the basis of an external examination [8]. Green turtles in the Galapagos have the slowest reported growth rate for this species [26]. Although the smallest nesting female recorded for the archipelago is 60.7 cm CCL, two peaks for nesting females exist at 80 and 95 cm CCL [59]. Furthermore, laparoscopic gonad assessment of green turtles has shown size at maturity is highly variable [20]. Therefore, we used a relatively large size of 80 cm CCL as a threshold to distinguish immature from mature individuals. Adults were sexed on the basis of the external sexual dimorphism of adult green turtles, while some smaller individuals could also be sexed as males by their evident secondary sexual characteristics [54], [57]. An EBRO^®^ Compact J/K/T/E Thermocouple Thermometer was used to obtain all temperature readings (model EW-91219-40; Cole-Parmer, Vernon Hills, Illinois, USA 60061). Core body temperatures were recorded from the cloaca using the probe T PVC epoxy tip 24GA×3 ft in length.

### Statistical Analysis

First, the iSTAT blood chemistry results of all overlapping values of the CG8+ and CG4+ cartridges (all analytes except lactate) were compared using paired t-tests. The iSTAT results for hematocrit were compared (paired t-test) to the results of manually determined hematocrit. Subsequently, we grouped values for each blood analyte by foraging site and compared them using Student’s t-test. Group sizes for different age classes (mature/immature) as well as sex (male/female) were too small to perform statistical analysis. Linear regressions were used to examine the relationship between the turtles’ body size or body temperature and the measured blood chemistry analytes. A standard alpha level of p = 0.05 was used for all statistical tests using R statistical software, version 3.0.2 (R Development Core Team).

## Results

### Turtle Demographics and Health Status

A total of 28 green turtles, 23 immature (CCL<80 cm) and 5 mature (CCL>80 cm) were sampled. Half of the animals (n = 14) were captured at each study location. The mean CCL was 68.0 cm; values ranged from 41.8 cm CCL for the smallest immature to 84.6 cm CCL for the largest adult male. All of the mature sea turtles (n = 5) were males and an additional three males of 79, 76.5 and 71.1 cm CCL were identified. Internal body temperature varied little among individuals, with a mean of 20.6°C and ranging between 19.4°C and 22.5°C. All 28 sea turtles appeared clinically healthy, had no barnacle or excessive algae growth on their carapaces and were bright, alert, and responsive. Many were observed feeding just prior to capture. One turtle had a large piece of monofilament fishing line on the right front flipper causing a strangulating lesion at the elbow that was deemed significant but had not compromised blood flow to the distal limb. The ligature was cut free of the turtle and discarded.

### Blood Biochemical Analysis

Values obtained from the CG4+ and CG8+ cartridges were consistent and not statistically different; thus, all overlapping iSTAT values reported are from the first cartridge run for all samples (CG8+). Table 1 displays the biochemistry, blood gas, and hematology results. In a few samples the iSTAT blood analyzer was unable to calculate values for some of the parameters resulting in a slightly smaller n. The hematocrit figures generated by the iSTAT were not significantly different from the manually determined values. There were no statistical differences between the two sampling sites for any values of blood gas, electrolyte, biochemical, and hematology parameters. In addition, none of these values, with one exception, were correlated either with body size or body temperature.

**Figure 2:**
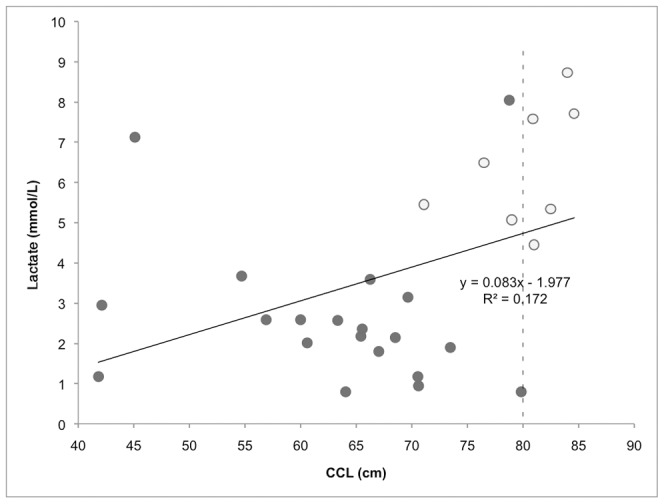
Scatter plot with linear regression line showing the correlation between sea turtle body size and blood lactate levels. Closed circles indicate data for immature turtles of undetermined sex. Open circles indicate turtles that could be identified as male based on secondary sexual characteristics. The dotted line indicates the size threshold (80 cm CCL) used to classify individuals as immature or mature.

The only blood analyte found to be positively correlated (r^2^ = 0.172, p<0.05) with body length (CCL) was lactate (Figure 2). Male green turtles appeared to have a higher lactate concentration than the rest of the unsexed and immature animals, but low sample size and uncertainty of determining sex of smaller individuals prevented a comparative analysis.

## Discussion

Previous studies have reported baseline blood gas, biochemical, and hematology parameters for some chelonian species, including sea turtles. Clinicians desire species-specific baseline values for the parameters commonly measured by commercial blood gas and chemistry analyzers. When working with reptiles, species specificity of health data is especially important, due to the diverse environmental conditions that exist in different habitats. This study reports the first set of blood gas, biochemistry, and hematology values in Galapagos green turtles and, indeed, in any subequatorial Western Hemisphere green turtle population.

Field sampling of green turtles was performed after the nesting season, when a resident subpopulation appears to remain in the Galapagos [16], [26], [48]. In addition, the two foraging areas sampled are frequented by resident turtles, some of which have been observed repeatedly for up to 5 years [16]. Thus, the turtles sampled in our study were most likely resident turtles, although the possibility that some of the turtles were transient visitors rather than residents cannot be ruled out.

The small sample size (n = 28) in the present study precluded the calculation of formal reference intervals, a process that would require a minimum of 120 individuals [23]. However, given the limited sample size available, and the lack of previously published data regarding biochemical, blood gas, and hematology parameters in resident Galapagos green turtles of the eastern tropical Pacific subspecies, these results provide a useful starting point for clinicians and researchers. All 28 turtles were judged to be clinically healthy and their blood parameters support this assessment.

In general, most of the blood parameters we recorded were similar to those reported previously for healthy green turtles [1], [17], [22], [30], [40]. For example, Harms et al. (2009) [30] reported iSTAT values obtained from five green turtles kept in captivity for several years. Relative to these, Galapagos green turtles had slightly lower pO_2_, higher pCO_2_, and slightly lower lactate**.** Similarly**,** Anderson et al. (2009) [5] reported median values for green turtles in North Carolina, U.S.A.; relative to the Galapagos turtles, most values were nearly equivalent, but significant differences existed in hematocrit and blood glucose. The hematology values we observed appear consistent with ranges reported for other clinically healthy green turtles and other chelonian species [1], [5], [8], [17], [25], [45], [56], [58].

In our study, parameter values affected by temperature were corrected based on published, standard formulas; these corrected values sometimes differed from auto-corrected iSTAT values (Table 1) but the differences did not appear to be clinically important. We conclude that iSTAT auto-corrected values are usually sufficient for clinical applications in the field, but also suggest that when accuracy is paramount, investigators studying animals with body temperatures below 37°C should be cautious about relying on auto-corrected iSTAT values for parameters that are influenced by temperature.

Galapagos green turtles in general had higher lactate values than other sea turtles [29], [32], [51], [52]. Only gillnet trapped green turtles [50], loggerhead turtles captured in shrimp trawlers, and cold-stunned Kemp’s ridley turtles (sampled after 2 to 3 days of hospitalization) [28], [36], had higher lactate. Loggerheads caught using pound nets had low lactate levels when sampled initially, but lactate concentrations were significantly higher after 30 min [28]. Similarly, Berkson (1966) [7] found no increase in blood lactate of green turtles until 30 to 60 min after forced submergence. Since most voluntary dives are thought to be aerobic and no difference has been found in the dive time of green turtles differing in size, none of these factors readily explain the overall high blood lactate and its increase with body size [41], [49]; Figure 2.

Several factors can influence chelonian biochemistry values, including environmental conditions, age, and sex. Relative to males, females frequently possess higher albumin, calcium, cholesterol, phosphorus, and triglyceride values, which are generally attributed to vitellogenesis [9], [14], [46], [58]. We could not investigate differences between sexes with regard to calcium or other parameters in the Galapagos green turtles due to the difficulty of determining the sex of immature animals in the field [53].

Age also affects some biochemical parameters in sea turtles. Casale et al. (2009) [12] reported that juvenile loggerhead turtles had lower values of albumin, calcium, globulins, hematocrit, total protein, and triglycerides than did adult animals, a difference probably at least partly attributable to egg production in adult females. In green turtles, albumin, total protein, and triglyceride levels were found to increase with body size while calcium, glucose, potassium, and sodium did not [39].

A number of papers have examined differences between clinically healthy and ill or compromised green sea turtles. Aguirre et al. (2009) [1] determined, by examining hematology, biochemistry, and adrenocortical values, that green turtles afflicted with green turtle fibropapillomatosis (GTFP) were immunocompromised and stressed. Similarly, Anderson et al. (2011) [5] reported that many important biochemical parameters (including calcium, glucose, total protein, and some electrolytes) were significantly lower, and uric acid and blood urea nitrogen higher, in cold-stunned green turtles versus healthy, wild individuals.

In a recent study with a very large sample size, Flint et al. (2010) [18] report blood reference intervals (RIs) for 290 Australian green turtles, 211 of which were deemed clinically healthy. All of the 25 turtles judged unhealthy possessed at least one value outside of the RIs. Aside from higher blood glucose levels in the Australian turtles, healthy green turtles in Australia and the Galapagos appear to have similar blood parameters.

In summary, data reported in this study represent an important step toward determining the normal range of values against which future blood gas and biochemistry results in green turtles can be compared. Such assessments are important for health monitoring and disease diagnostics. Because green turtles are an endangered species with importance in the wildlife biology research community and the aquarium/zoo industry, health assessments are important from the standpoint of sustainable conservation and management. These results add to a growing database of knowledge about health management in wild chelonian species. Future research should continue to establish reference values in this species and facilitate comparisons of blood values across age groups and disease states.
